# Damage to sebaceous gland and the efficacy of moisturizer after whole breast radiotherapy: a randomized controlled trial

**DOI:** 10.1186/s12885-019-5334-9

**Published:** 2019-02-07

**Authors:** Mami Ogita, Kenji Sekiguchi, Keiko Akahane, Ryoko Ito, Chiori Haga, Satoru Arai, Yasushi Ishida, Jiro Kawamori

**Affiliations:** 1 Department of Radiation Oncology, St. Luke’s International Hospital, 9-1 Akashi-cho, Chuo-ku, Tokyo, 104-8560 Japan; 2 Department of Radiology, The University of Tokyo Hospital, 7-3-1 Hongo, Bunkyoku, Tokyo, 113-8655 Japan; 3 Sonoda-kai Radiation Oncology Clinic, 3-4-19, Hokima, Adachi-ku, Tokyo, 121-0064 Japan; 4 Department of Radiology, Saitama Medical Center Jichi Medical University, 1-847 Amanuma-cho, Omiya-ku, Saitama, 330-8503 Japan; 5 Department of Dermatology, St. Luke’s International Hospital, 9-1 Akashi-cho, Chuo-ku, Tokyo, 104-8560 Japan; 6 Center for Clinical Epidemiology, St. Luke’s International Hospital, 3-6-2 Tsukiji, Chuo-ku, Tokyo, 104-0045 Japan

**Keywords:** Sebaceous gland damage, Radiotherapy, Breast cancer, Moisturizer

## Abstract

**Background:**

We conducted a randomized trial to evaluate the efficacy of heparinoid moisturization for radiation dermatitis. We report the time-course of sebum content after whole breast radiotherapy (WBRT) and the efficacy of heparinoid moisturizer.

**Methods:**

Patients receiving adjuvant breast RT were randomly assigned into three groups; prophylaxis, post-WBRT and control groups. Patients used moisturizer on the irradiated breast from the beginning of RT in the prophylaxis group, 2 weeks post-RT in the post-WBRT group, and no moisturizer in the control group. Sebum content of the irradiated and non-irradiated breast was measured to assess sebaceous gland damage. Sebum composition was also analyzed.

**Results:**

A total of 76 patients were analyzed; 30 in the post-WBRT group, 32 in the control group, 14 in the prophylaxis group. The sebum content in the irradiated breast significantly decreased after WBRT in the post-WBRT and control groups. The decrease was sustained in the control group. In the non-irradiated breast, sebum content also decreased after WBRT in the post-WBRT and control groups. After moisturizer application, sebum content by sebumeter returned to pre-RT level in the post-WBRT group, while the decrease was sustained in the control group. Sebum content measured by evaporative light scattering detector and sebumeter was similar in the control group, but the dissociation was observed after moisturizer application in the post-WBRT group. The proportion of wax esters decreased in the irradiated breast after WBRT.

**Conclusions:**

Radiotherapy significantly reduced sebum content in both irradiated and non-irradiated breast, indicating that RT caused quantifiably persistent sebaceous gland damage in irradiated sites and the surrounding tissue. Combined with the results from our previous study, heparinoid moisturizer treatment effectively prevents water loss by retaining oil contents on the skin surface.

**Trial registration:**

UMIN, UMIN000005532. Registered 1 April 2011.

**Electronic supplementary material:**

The online version of this article (10.1186/s12885-019-5334-9) contains supplementary material, which is available to authorized users.

## Background

The whole breast radiotherapy (WBRT) after breast-conserving surgery reduces the risk of recurrence and death and is widely used for standard treatment for breast cancer. [1, 2] Radiation dermatitis is a major adverse event of WBRT. [3] Radiotherapy (RT) damages skin structure and causes a variety of symptoms. [4] Sebaceous gland is a part of skin appendages and has an important role for skin protection. [5] Sebum delivers antioxidants to the skin surface and prevents the buildup of reactive oxygen species which causes a breakdown of the skin barrier. [6] Destruction of sebaceous and sweat glands can lead to skin dryness and affect the patient’s quality of life (QOL). Although the sebaceous gland is considered to be more radiosensitive than the sweat gland, [7–9] few clinical data are available to demonstrate this.

Several studies have been published to show the efficacy of topical skin agents for the prevention or reduction of radiation dermatitis, but most of them failed to show effectiveness. [4, 10, 11] We therefore conducted a randomized trial to evaluate the efficacy of heparinoid moisturizer for the prevention and reduction of radiation dermatitis. Heparinoid moisturizer contains mucopolysaccharide polysulphate as an active substance, which has hydrophilic structures and creates bonding with adjacent water molecules. This leads to hydration of the stratum corneum. [12] Heparinoid moisturizer effectively improves dry skin and is widely used for its treatment in our country. [13] We measured the water content (WC) of stratum corneum and sebum content prospectively to assess skin damage following RT.

We previously reported the time-course of WC of stratum corneum after WBRT and showed that heparinoid moisturizer effectively increased the WC of stratum corneum and the preventive application reduced skin desquamation and dryness. [14, 15] In the present article, we report the time-course of sebum content and the efficacy of heparinoid moisturizer.

## Methods

### Study design and patients

This study was designed as a single-center, open-label, randomized controlled trial. Eligible patients were women aged 30 to 65 years with non-inflammatory breast cancer treated by breast-conserving surgery, and no surgical resection or boost in the inner-upper quadrant, which was designated as a measurement site. Patients were excluded if they had bilateral breast cancer, previous RT to the thorax, wide-spreading skin disease, collagen vascular disease, and sensitivity to heparinoid substance.

### Randomization

Patients were randomly assigned (1:4) to receive moisturizer (prophylaxis) or no moisturizer before WBRT. At two weeks after WBRT, patients in the no moisturizer group were randomly reassigned (1:1) to receive moisturizer (post-WBRT) or no moisturizer (control). At the second randomization, patients were stratified according to the relative ratio of skin WC on the last day of WBRT.

### Procedures

All patients received three-dimensional RT to the whole breast using the field-in-field technique. The dose to the whole breast was 48–50 Gy in 24–25 fractions. The supraclavicular region (50 Gy in 25 fractions) and/or boost to the tumor bed (10–18 Gy in 5–9 fractions) were given when required. Participants were not allowed to use the moisturizer until randomization. In the prophylaxis group, patients used moisturizer (heparinoid; Hirudoid®, Maruho, Japan) twice daily on their irradiated breast on the first day of WBRT and continued it during the study period. In the no moisturizer group, patients were instructed not to use moisturizer until two weeks after WBRT. At two weeks after WBRT, patients assigned to the post-WBRT group initiated moisturizer use. Patients in the control group were instructed not to use moisturizer throughout the study period. All patients were allowed to use topical corticosteroids when required. An adherence rate of 60% was set to the lower limit for acceptance.

### Measurement

Patients were instructed not to bathe in the morning and not to apply any products to the bilateral breast on the measurement day. A minimum of 20 min bed rest was required before the measurement to avoid the effect of the outside environment. The measurement areas were 3 × 3 cm^2^ skin surface in an upper-inner quadrant of the irradiated and contralateral non-irradiated breast, 2 cm apart from the midline, and at least 2 cm away from the surgical wound and 1 cm away from the boost edge. For additional analysis, sebum of middle and outer area of the non-irradiated breast was collected in part of patients in the control group (Additional file 1 Figure S1). Sebum content was measured by the Sebumeter® (Courage+Khazaka electronic GmbH) four times at different points in the same area at baseline, last day of WBRT, 2 weeks, 4 weeks, and 3 months post-RT. When the patient agreed, measurements were performed at 6, 9, and 12 months post-RT. The mean value of sebum content was used for the analysis. The Sebumeter® is widely used in medical research for measuring sebum content. The tape was placed on the skin, and the transparency is measured by a photocell. The light transmission represents the sebum content. [16, 17] Sebum composition and content were analyzed by chromatography (HP1100 Agilent Technologies) and evaporative light scattering detector (ELSD, SofTA 300SM&S Instruments Inc.). Analysis of the sebum composition by ELSD was performed as they could potentially provide additional useful information. Sebum is composed of five classes of lipid; triglyceride, a wax ester, squalene, free fatty acid, and cholesterol. [6, 18, 19] The sebum amount was taken as the sum of the five lipids. Sebum was collected in the no moisturizer group at baseline. After the second randomization, sebum was collected in the control group at 2 weeks and 3 months post-RT. For additional exploration, sebum of 4 patients in the post-WBRT group was analyzed.

### Outcome

Sebum content and sebum composition were pre-defined secondary endpoints in this study. The primary endpoint of the WC of stratum corneum and the other secondary outcomes of signs and symptoms associated with acute radiation dermatitis were previously reported. [14, 15] In this article, we report the time-course of sebum.

### Statistical analysis

Sample size calculations for the primary endpoints were reported previously. [14] For the comparison of patient characteristics, the Chi-squared test and Kruskal-Wallis test were used. For the comparison of sebum content, the Wilcoxon signed-rank test with Bonferroni’s correction and Mann-Whitney U test were used. All tests were two-sided and *P* value < 0.05 was considered statistically significant. Statistical analyses were performed using SPSS (IBM SPSS statistics 21; IBM, NY, USA).

This study was approved by the institutional review board of St. Luke’s International Hospital (11-R060) and registered with UMIN000005532. Written informed consent was obtained from each patient before enrollment.

## Results

### Baseline characteristics

**Fig. 1:**
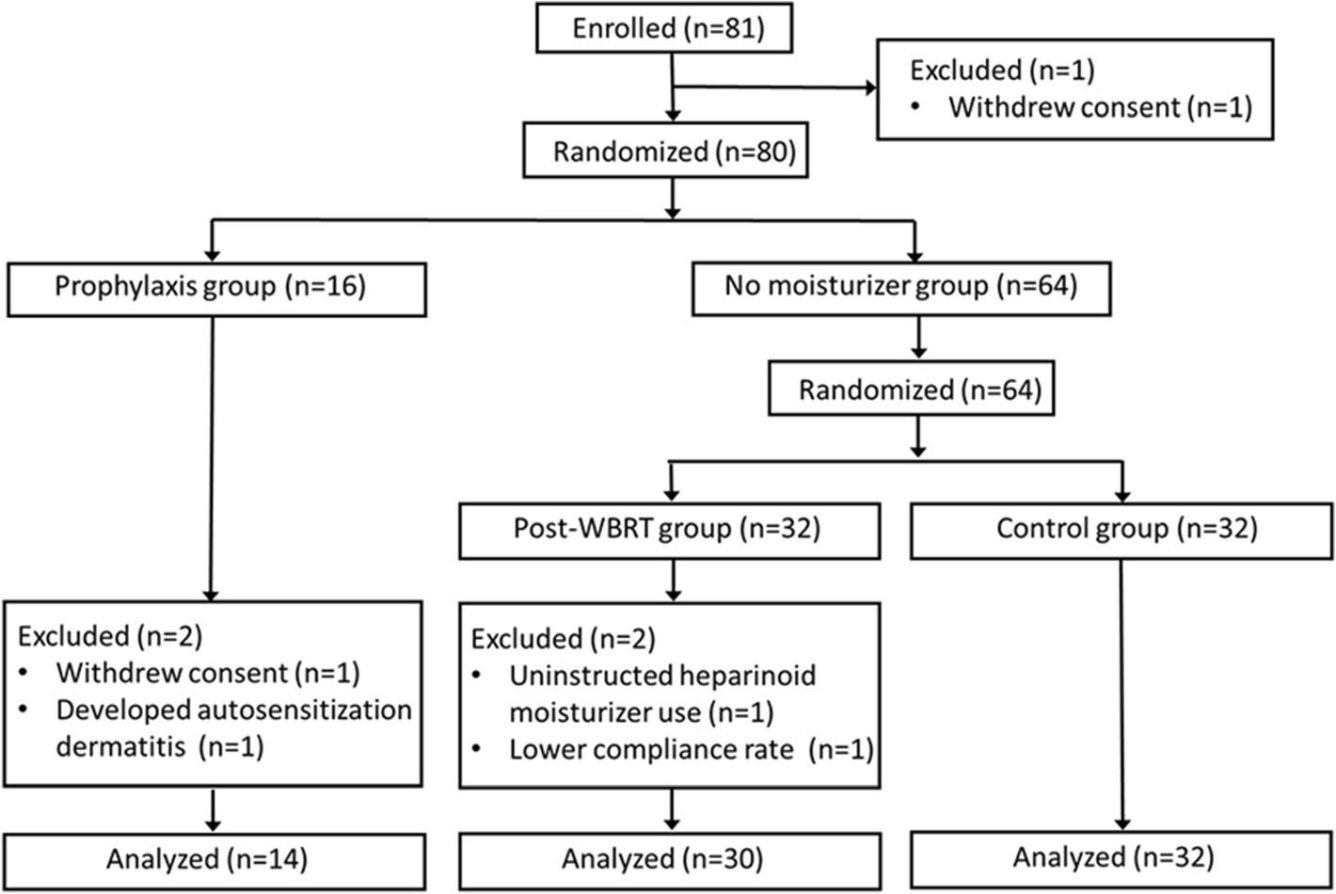
The Consort flow diagram. WBRT; whole breast radiotherapy

Between April 2011 and April 2013, 81 patients were enrolled. One patient withdrew consent immediately after enrollment. After randomization, four patients were excluded from analysis for the following reasons; withdrew consent (*n* = 1), uninstructed heparinoid moisturizer use (n = 1), lower compliance rate (n = 1), developed autosensitization dermatitis (n = 1). A total of 76 patients were analyzed; 14 patients in the prophylaxis group, 30 patients in the post-WBRT group and 32 patients in the control group (Fig. 1). Baseline characteristics are listed in Table 1, and were well balanced between the three groups.

**Table 1:** Patient baseline characteristics

	Prophylaxis (*n* = 14)		Post WBRT (*n* = 30)		Control (*n* = 32)		*p*
	*n*	%	*n*	%	*n*	%	
Age (year)							
Median (range)	45 (34–65)		49 (32–65)		50 (39–65)		0.13
BMI							
Median (range)	21.3 (17–28)		22.3 (17–33)		20.9 (16–29)		0.28
Affected breast							
Left	8	57	18	60	14	44	0.4
Right	6	23	12	40	18	56	
Tumor location							
Inner	0	0	2	7	1	3	0.24
Lateral	13	93	28	93	29	91	
Central	1	7	0	0	2	6	
Smoking							
Current	0	0	0	0	1	3	0.74
Past	4	29	6	20	6	19	
Never	10	71	24	80	25	78	
Chemotherapy before RT							
Yes	4	29	5	83	4	13	0.41
No	10	71	25	17	28	87	
Endocrine therapy before RT							
Yes	1	7	2	7	1	3	0.77
No	13	93	28	93	31	97	
RT							
Energy (MV)							
4	10	72	18	60	24	75	0.43
6	4	29	12	40	8	25	
Boost							
10–18 Gy	9	64	15	50	16	50	0.63
No	5	36	15	50	16	50	

BMI; body mass index, RT; radiotherapy, WBRT; whole breast radiotherapy

### Sebum content by sebumeter

#### Irradiated breast

**Fig. 2:**
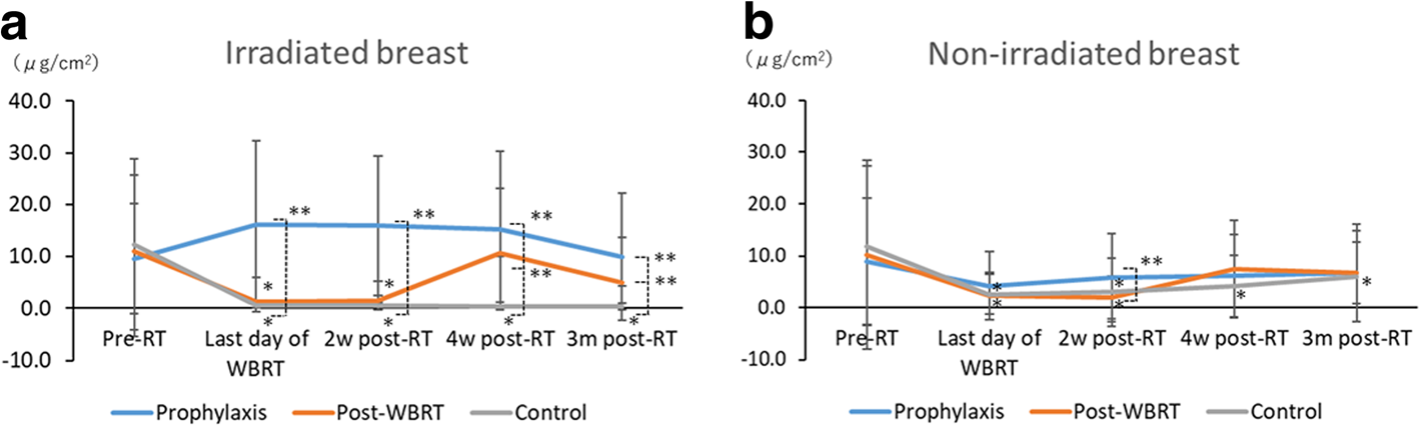
Time course of sebum content by sebumeter in the irradiated (**a**) and non-irradiated breast (**b**). a * *P* <  0.05 comparison between baseline and each time point. ** *P* <  0.05 comparison between control group and each group. RT; radiotherapy; WBRT; whole breast radiotherapy. b * *P* < 0.05 comparison between baseline and each time point. ** *P* < 0.05 comparison between control group and each group. RT; radiotherapy; WBRT; whole breast radiotherapy

At baseline, the mean sebum content of irradiated breast was similar between the three groups (9.6 ± 10.6 μg/cm^2^ in prophylaxis, 11.1 ± 14.6 μg/cm^2^ in post-WBRT and 12.3 ± 16.5 μg/cm^2^ in control). In the post-WBRT and control groups, the mean sebum content of irradiated breast was significantly decreased following WBRT on the last day of WBRT (1.3 ± 4.6 μg/cm^2^ in post-WBRT and 0.5 ± 1.2 μg/cm^2^ in control) and 2 weeks post-RT (1.5 ± 3.8 μg/cm^2^ in post-WBRT and 0.5 ± 0.7 μg/cm^2^ in control) (*P* <  0.001; *P* <  0.001, respectively). After applying moisturizer, sebum content in the post-WBRT group returned to pre-RT levels at 4 weeks (10.7 ± 12.4 μg/cm^2^) and 3 months post-RT (4.9 ± 8.8 μg/cm^2^) (*P* = 1.0; *P* = 0.08, respectively), while reduction was sustained in the control group (0.4 ± 0.7 μg/cm^2^ and 0.4 ± 0.6 μg/cm^2^ at 4 weeks and 3 months post-RT, respectively) (*P* <  0.001; *P* <  0.001, respectively). In the prophylaxis group, the mean sebum content of irradiated breast remained at pre-RT levels throughout the study period (16.2 ± 16.1 μg/cm^2^, 16.0 ± 13.4 μg/cm^2^, 15.2 ± 15.1 μg/cm^2^, 10.0 ± 12.3 μg/cm^2^ on the last day, at 2 weeks, 4 weeks, and 3 months post-RT, respectively) (*P* = 1.0; *P* = 0.88; *P* = 1.0; *P* = 1.0, respectively) (Fig. 2a).

Sebum content significantly decreased in the post-WBRT and control groups vs. the prophylaxis group on the last day of WBRT and 2 weeks post-RT (*P* <  0.001; *P* <  0.001, respectively). The significant decrease was sustained to 3 months in the control group. Moisturizer use kept or returned sebum content at pre-RT levels (Table 2a).

**Table 2:** Sebum content by sebumeter in the irradiated (a) and non-irradiated breast (b)

	Prophylaxis (*n* = 14)	vs Control	Post-WBRT (*n* = 30)	vs Control	Control (*n* = 32)
	Mean (μg/cm^2^) (± SD)	*P*	Mean (μg/cm^2^) (± SD)	*P*	Mean (μg/cm^2^) (± SD)
a. Irradiated breast					
Pre-RT	9.6 (± 10.6)	0.66	11.1 (± 14.6)	0.5	12.3 (± 16.5)
Last day of WBRT	16.2 (± 16.1)	< 0.01	1.3 (± 4.6)	0.87	0.5 (± 1.2)
2w post-RT	16.0 (± 13.4)	< 0.01	1.5 (± 3.8)	0.81	0.5 (± 0.7)
4w post-RT	15.2 (± 15.1)	< 0.01	10.7 (± 12.4)	< 0.01	0.4 (± 0.7)
3 m post-RT	10.0 (± 12.3)	< 0.01	4.9 (± 8.8)	0.01	0.4 (± 0.6)
b. Non-irradiated breast					
Pre-RT	8.9 (± 12.2)	0.52	10.2 (± 18.2)	0.34	11.9 (± 15.4)
Last day of WBRT	4.2 (± 6.6)	0.45	2.2 (± 4.5)	0.27	2.6 (± 3.9)
2w post RT	5.8 (± 8.5)	0.03	1.9 (± 4.1)	1	3.0 (± 6.5)
4w post RT	6.2 (± 7.9)	0.15	7.4 (± 9.5)	0.08	4.1 (± 6.0)
3 m post RT	6.8 (± 5.9)	0.23	6.7 (± 9.5)	0.75	6.0 (± 8.8)

RT; radiotherapy, WBRT; whole breast radiotherapy

#### Non-irradiated breast

At baseline, the mean sebum content of non-irradiated breast was similar among the three groups (8.9 ± 12.2 μg/cm^2^ in prophylaxis, 10.2 ± 18.2 μg/cm^2^ in post-WBRT and 11.9 ± 15.4 μg/cm^2^ in control), and no difference was found between the irradiated and non-irradiated breast. The mean sebum content of non-irradiated breast was also significantly decreased following WBRT up to 2 weeks post-RT in both post-WBRT (1.9 ± 4.1 μg/cm^2^) and control groups (3.0 ± 6.5 μg/cm^2^) (*P* <  0.001; *P* <  0.001, respectively). Sebum content returned to pre-RT levels at 4 weeks (7.4 ± 9.5 μg/cm^2^) and 3 months post-RT (6.7 ± 9.5 μg/cm^2^) in the post-WBRT group (*P* = 1.0; *P* = 1.0, respectively). The significant decline was sustained during the study period in the control group (*P* = 0.002; *P* = 0.049 at 4 weeks and 3 months post-RT, respectively). In prophylaxis group, the mean sebum content of the non-irradiated breast was not significantly different from pre-RT levels through the study period (*P* = 0.35; *P* = 1.0; *P* = 1.0; *P* = 1.0, on the last day, at 2 weeks, 4 weeks, and 3 months post-RT, respectively). (Fig. 2b).

Sebum content significantly decreased in the post-RT and control groups vs. the prophylaxis group at 2 weeks post-RT (*P* = 0.03). There were no significant differences in sebum content between the three groups at the other time points studied (Table 2b).

#### Longer follow-up

In the control group, sebum content was measured in 9 patients at 6 months post-RT and 5 patients at 9 and 12 months post-RT. In the irradiated breast, the decrease of sebum content was sustained for up to 12-month post-RT (12.3 ± 16.5 μg/cm^2^ at pre-RT vs. 0.05 ± 0.1 μg/cm^2^ at 12 months post-RT, *P* = 0.04). In the non-irradiated breast, sebum content decreased, but no statistical difference was observed after 4 weeks post-RT (11.9 ± 15.4 μg/cm^2^, 4.3 μg/cm^2^ ± 4.0 μg/cm^2^, 5.2 μg/cm^2^ ± 6.0 μg/cm^2^, 2.3 μg/cm^2^ ± 1.9 μg/cm^2^; *P* = 0.30; *P* = 0.23; *P* = 0.14, at pre-RT, 6 months, 9 months, and 12 months post-RT, respectively) (Additional file 1 Figure S2).

### Sebum composition and sebum content by ELSD

**Fig. 3:**
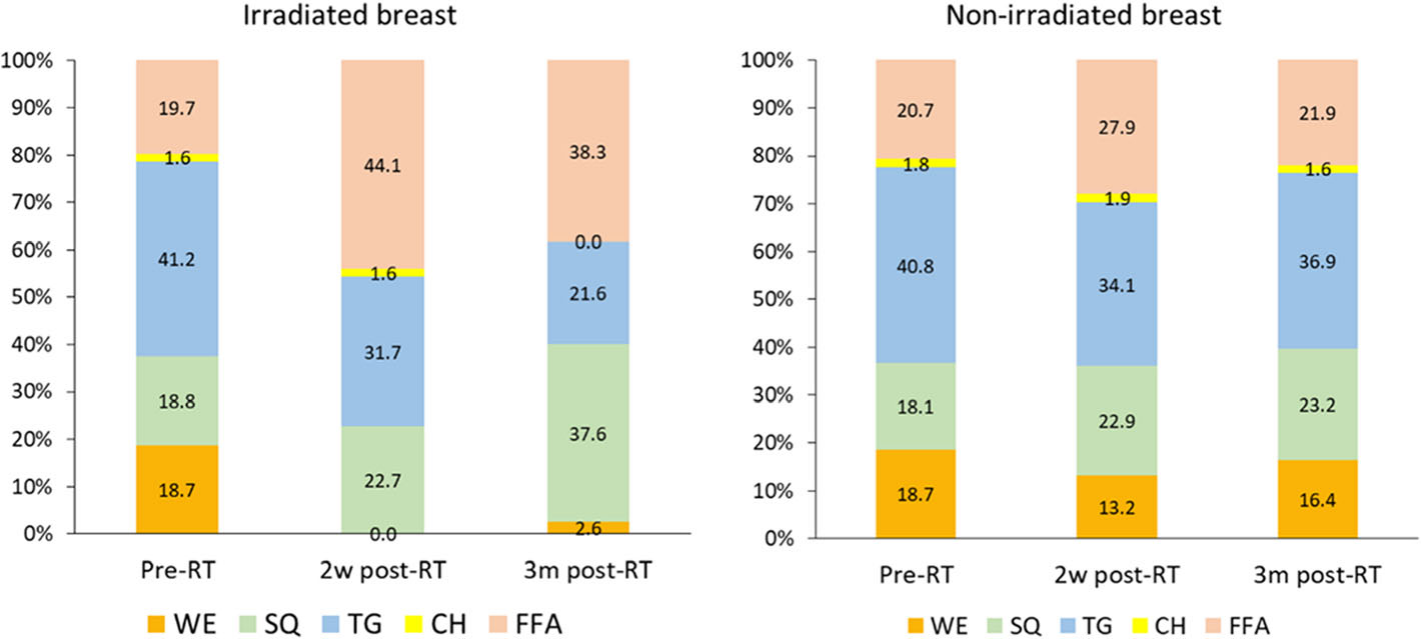
Sebum composition in the irradiated and non-irradiated breast of 15 patients in the control group. WE; Wax ester, SQ; Squalene, TG; Triglyceride, CH; Cholesterol, FFA; Free fatty acids

Sebum composition data were collected among 21 patients in the control group. Because baseline data of 6 patients was not available due to a technical issue, sebum composition of 15 patients was used for analysis. The proportion of wax esters, which is unique to sebum gland, decreased significantly from 18.7 ± 4.3% at baseline to 2.6 ± 8.3% at 3 months post-RT in the irradiated breast (*P* = 0.04). In the non-irradiated breast, the proportion of wax esters did not change (18.7 ± 4.3% at baseline to 16.4 ± 9.2% at 3 months post-RT; *P* = 0.78). The proportion of other sebum composition is provided in Fig. 3.

Sebum content by ELSD of the control group was significantly decreased in irradiated breast at 2 weeks (0.56 ± 0.62 μg/cm^2^) and 3 months post-RT (0.39 ± 0.46 μg/cm^2^) compared with baseline (13.0 ± 19.7 μg/cm^2^) (*P* = 0.001; *P* = 0.001, respectively). In non-irradiated breast, sebum content was decreased at 2 weeks post-RT (3.2 ± 2.5 μg/cm^2^) (*P* = 0.001) but returned to baseline levels (10.7 ± 13.7 μg/cm^2^) at 3 months post-RT (8.5 ± 17.0 μg/cm^2^) (*P* = 0.11). Sebum content by ELSD and sebumeter was similar in the control group and at 2 weeks post-RT in the post-WBRT group, but the dissociation was observed after moisturizer application in the post-WBRT group. Sebum content by sebumeter was much higher than sebum content by ELSD at 4 weeks and 3 months post-RT (Table S1).

Sebum content of inner area was decreased at 2 weeks post-RT, but sebum of middle and outer areas was not significantly different from pre-RT levels (Table 3).

**Table 3:** The influence of the distance from the irradiated field on sebum content by ELSD

	Irradiated area		Non-irradiated inner		Non-irradiated middle		Non-irradiated outer	*P*
	Mean (μg/cm2) (± SD)	*P*	Mean (μg/cm^2^) (± SD)	*P*	Mean (μg/cm^2^) (± SD)	*P*	Mean (μg/cm^2^) (± SD)	
Pre-RT (*n* = 7)	15.0 (± 26.2)		11.0 (± 18.5)		6.4 (± 1.0)		3.5 (± 6.5)	
2w post RT (n = 7)	0.08 (± 0.10)	0.02	2.7 (± 2.7)	0.02	1.6 (± 2.1)	0.09	1.3 (± 2.1)	0.13
3 m post RT (*n* = 6)	0.23 (± 0.33)	0.03	12.5 (± 24.0)	0.6	6.9 (± 13.2)	0.75	2.8 (± 5.0)	0.46

ELSD; evaporative light scattering detector, RT; radiotherapy

## Discussion

Sebum content was found in the present study to be significantly decreased after WBRT in the irradiated field and surrounding areas, and the reduction was sustained. To our knowledge, this is the first study to quantitatively evaluate the skin sebum contents after RT using an objective method.

Sebum has an important role in skin protection and stratum corneum hydration. [6, 20] In our study, sebum levels in the irradiated breast were almost zero without moisturizer application. Although the longer follow-up data were collected from a small amount of the patients, the reduction of sebum was sustained for up to 12 months after RT. Radiotherapy may cause persistent damage to the sebaceous gland.

Sebum content decreased significantly in the non-irradiated breast, even though no direct beam was irradiated in the contralateral breast. To estimate the absorbed dose in the contralateral breast, we measured the surface dose of non-irradiated measurement site using breast phantom. The measurement site on the contralateral breast was located at a specified distance away from the radiation field by approximately 2 cm (inner), 5 cm (middle), and 8 cm (outer). The estimated dose was 150 mGy/fr, 100 mGy, 60 m Gy per 2 Gy/fr prescription dose, respectively, which was most likely caused by scattered ray (data not shown). A previous study reported contralateral breast surface 2–12 cm from the midline received 2–12 Gy for a 50 Gy treatment. The contralateral breast was exposed by leakage radiation or scattered ray from the collimator or treated breast. [21] Although there are no reports on the tolerance dose of the sebaceous gland, it is considered to be more radiosensitive than the sweat gland. [7–9] Approximately 70% of patients who received equivalent total doses 42–46 Gy in 2 Gy fractions experienced sweat gland dysfunction. Most patients demonstrated the recovery of sweat gland function, but a few of them showed little recovery up to 9 months. [22] Our results indicate that sebaceous glands are much more sensitive than sweat glands because they were affected by very low doses, such as scattered rays, and that damage can last for up to 12 months.

Most of the skin surface lipids come from the sebaceous glands, with the other remaining lipids coming from the stratum corneum cells of the epidermis. [18, 23] Human sebum contains triglyceride (20–60%), wax ester (23–30%), squalene (10–20%), free fatty acid (5–40%), cholesterol (1–5%), and diglycerides (1–2%). [6, 18, 19, 24] Epidermal lipids contain triglyceride, diglycerides and free fatty acids (38–65%), wax ester (0%), squalene (< 0.5%), and cholesterol (20–25%). [6, 18, 19] Wax esters are unique to sebum and not produced at other organs in the body. [6, 20] The observed decrease in the level of wax esters in the irradiated field may be indicative of impairment of sebum secretion function.

The sebum content as measured by sebumeter returned to pre-RT level in the post-WBRT group after moisturizer application. To confirm the efficacy of heparinoid moisturizer on radiation-induced asteotosis, we measured the sebum content in the control and post-WBRT groups by ELSD. Sebum content by ELSD and sebumeter was similar in the control group, but the dissociation was observed after moisturizer application in the post-WBRT group. Sebum content by ELSD did not change, but sebum content by sebumeter increased after moisturizer application. Sebumeter measured the transparency of the lipid absorbed tape as a sebum (oil) content value, however, the sebum level by sebumeter may not reflect the exact function of the sebaceous gland. Although we instructed patients not to apply moisturizer on the measurement day, the sebumeter sebum value may have been affected by the presence of other oil contents on the skin surface from the moisturizer oil base, such as white petrolatum.

Sebum content in the non-irradiated breast had no statistical difference from the pre-RT level in the prophylaxis group and recovered to the pre-RT level after moisturizer application in the post-WBRT group. Patients were instructed not to apply heparinoid moisturizer to the outside of the irradiated field. However, some may have unintentionally applied the moisturizer outside of the irradiated field, because the measurement site in the non-irradiated breast was very close to the midline. This may have influenced the recorded measurement.

A variety of randomized controlled trials have been performed to show the effectiveness of topical therapy for radiation dermatitis, but little has shown the superiority of any agents. [4, 10, 11] A topical steroid cream for prophylactic use reduced the incidence of acute radiation dermatitis, and had a beneficial effect on patients’ QOL. [25–27] In our previous report, heparinoid moisturizer maintained stratum corneum WC and reduced skin desquamation and dryness. [14, 15] The skin itself is not the primary target of RT in breast-conserving therapy. [27] However, the skin exposure to significant doses is inevitable during RT, thereby causing damage to the sweat and sebaceous glands. The present results indicate that heparinoid moisturizer may be a suitable substitute for sebaceous and sweat gland secretion, providing the desired level of skin lubrication. Combined with the results from our previous study, heparinoid moisturizer application helps to maintain WC and prevents water loss by retaining oil contents. Heparinoid moisturizer therefore represents an ideal choice of topical treatment. As sebaceous gland damage occurred both within the irradiated field and to the surrounding tissue, we recommend applying heparinoid moisturizer to both areas.

There were several limitations to our study. Firstly, change in observed sebum content was a secondary endpoint. Due to the small study population, some of our results may not have enough power to show the difference. Secondly, as mentioned above, the sebum level measured by sebumeter may not reflect the exact function of the sebaceous gland.

To further enhance our knowledge, we are currently conducting a new randomized controlled trial to evaluate the effect of heparinoid moisturizer on skin-related QOL by using Dermatology Life Quality Index [28] (UMIN000026987).

## Conclusions

Radiotherapy significantly reduced sebum content in both the irradiated and non-irradiated breast, indicating that RT caused quantifiably persistent sebaceous gland damage at irradiated sites and the surrounding tissue. Sebum glands are very sensitive and are affected by very low doses, such as scattered rays. Heparinoid moisturizer treatment effectively prevents water loss by retaining oil contents on the skin surface.

## Additional file

Additional file 1: **Figure S1.** Measurement sites. **Figure S2.** Longer follow-up of sebum content by sebumeter in the irradiated (a) and non-irradiated breast (b) of the control group. Table S1 Comparison of sebum content measured by ELSD and sebumeter. (DOCX 359 kb)

## Abbreviations

ELSD: evaporative light scattering detector
QOL: quality of life
RT: radiotherapy
WBRT: whole breast radiotherapy
WC: water content
