# Supplementary material for: Damage to sebaceous gland and the efficacy of moisturizer after whole breast radiotherapy: a randomized controlled trial
Source: BMC Cancer. 2019 Feb 7;19:125. doi: 10.1186/s12885-019-5334-9 (PMC6367742; doi:10.1186/s12885-019-5334-9)
Supplement: Supplementary file 1 — Figure S1. Measurement sites. Figure S2. Longer follow-up of sebum content by sebumeter in the irradiated (a) and non-irradiated breast (b) of the control group. Table S1 Comparison of sebum content measured by ELSD and sebumeter. (DOCX 359 kb) [file 12885_2019_5334_MOESM1_ESM.docx]

Figure S1. Measurement sites


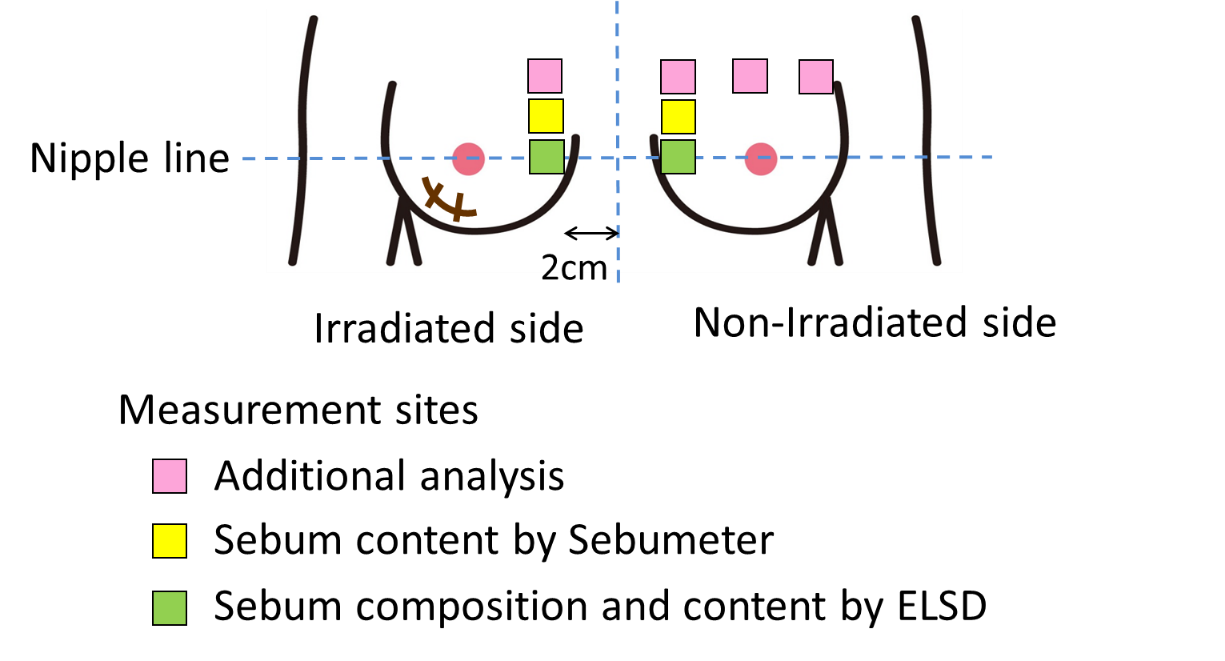


ELSD; evaporative light scattering detector

Figure S2. Longer follow-up of sebum content by sebumeter in the irradiated (a) and non-irradiated breast (b) of the control group

a


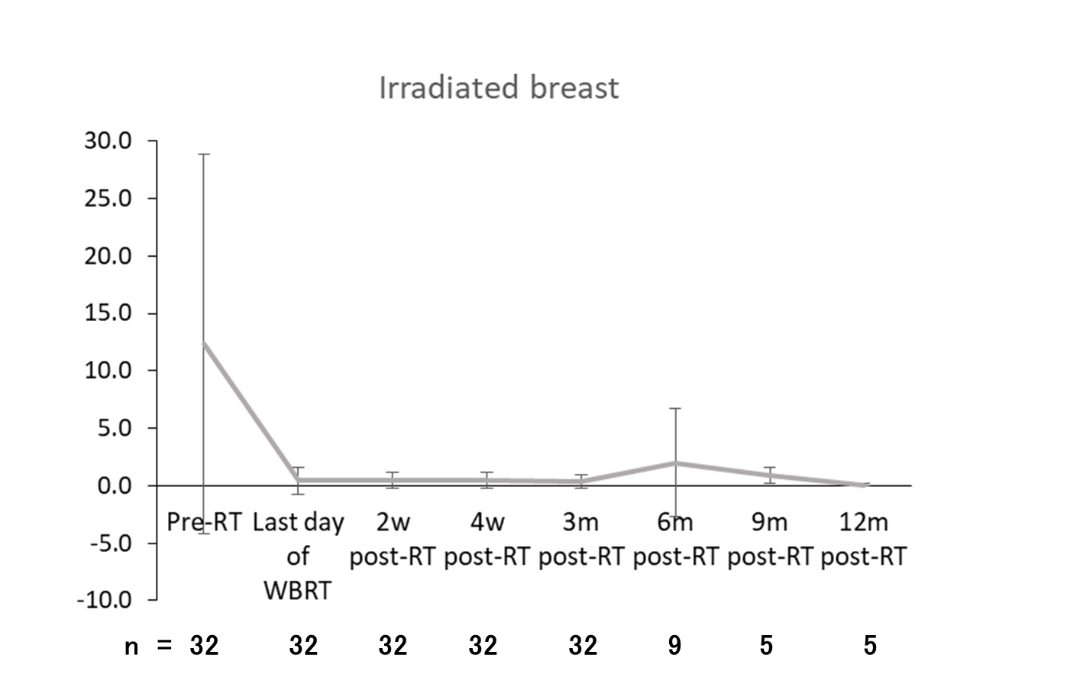


b


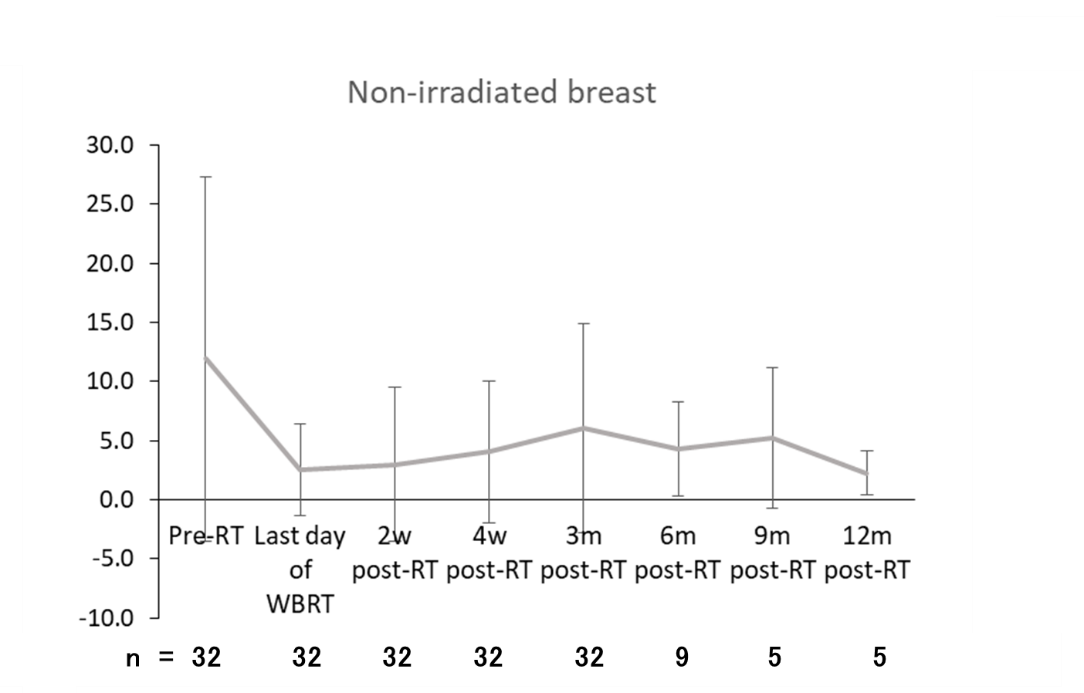


RT; radiotherapy; WBRT; whole breast radiotherapy

Table S1. Comparison of sebum content measured by ELSD and sebumeter

|  |  |  |  |
| --- | --- | --- | --- |
| Group | Time point | ELSD | Sebumeter |
|  |  | Mean (μg/cm^2^) (± SD) | Mean (μg/cm^2^) (± SD) |
| Control | Pre-RT | 13.0 (± 19.7) | 16.0 (± 21.3) |
| (n=15) | 2w post-RT | 0.56 (± 0.62) | 0.57 (± 0.90) |
|  | 3m post-RT | 0.39 (± 0.46) | 0.33 (± 0.68) |
| Post WBRT | 2w post-RT | 0.43 (± 0.41) | 1.4 (± 1.9) |
| (n=4) | 4w post-RT | 0.55 (± 0.71) | 8.2 (± 0.9) |
|  | 3m post-RT | 0.92 (± 0.66) | 15.0 (± 13.8) |
|  |  |  |  |

ELSD; evaporative light scattering detector, RT; radiotherapy; WBRT; whole breast radiotherapy
